# Improving the methodology for the detection of proviral integration sites in the host genome via high throughput sequencing

**DOI:** 10.1186/1742-4690-12-S1-P51

**Published:** 2015-08-28

**Authors:** Keith Durkin, Maria Artesi, Nicolas Rosewick, Ambroise Marçais, Olivier Hermine, Michel Georges, Anne Van den Broeke

**Affiliations:** 1 Unit of Animal Genomics, GIGA, Université de Liège, 40 Liège, Belgium; 2 Laboratory of Experimental Hematology, Université Libre de Bruxelles, 10 Brussels, Belgium; 3 Service d'Hématologie adultes, INSERM U1163 CNRS ERL 8254, Hôpital Universitaire Necker, Université René Descartes, Institut Imagine, Paris, France

## 

Bovine Leukemia Virus (BLV) and the closely related Human T-cell leukemia virus-1 (HTLV-1) are deltaretrovirus that induce leukemia/lymphoma in about ~5% of infected individuals. The mechanisms responsible for cellular transformation have remained largely enigmatic as both viruses are largely transcriptionally silent in tumors and show multiple integration sites in the host genome. The recent application of high throughput sequencing to track proviral insertion sites in the host genome has provided a number of insights into the evolution of deltaretrovirus infections and the progression of tumor clones in deltaretrovirus induced leukemia/lymphoma. However the protocols currently utilised have a number of limitations, including relatively high sequencing costs, the use of custom sequencing primers, no examination of the region upstream of the provirus and limited dynamic range for determining clone abundance. We have developed an alternative high throughput sequencing protocol for identifying proviral integration sites in BLV and HTLV-1 infected individuals that uses off-the-shelf Illumina primers for the addition of adapters and indexes. This greatly simplifies the process of multiplexing libraries and does away with the need for custom sequencing primers. Additionally our approach assays the region upstream of the provirus in addition to the downstream region, giving additional information on the frequency of 5’ deletions in proviruses and increasing the dynamic range of the assay. We have tested the approach on over 1 BLV and HTLV-1 samples, representing both tumors and preleukemic stages. Our approach allowed for a more accurate determination of clone abundance in tumors and by assaying the 5’ end of the provirus identified clones overlooked with previously published methods. Finally, by facilitating greater multiplexing of libraries we have reduced the cost to a level where the technique may be attractive in a clinical setting.

